# Strengthening pandemic prevention, preparedness, and response in Somalia: strategic implications of health security financing

**DOI:** 10.3389/fpubh.2026.1885373

**Published:** 2026-07-07

**Authors:** Abdullahi Ahmed Tahlil, Nor Ali Mohamoud, Ali Haji Adam, Abdinasir Yusuf Osman

**Affiliations:** 1 Ministry of Health and Human Services, Federal Government of Somalia, Mogadishu, Somalia; 2 Department of Public Health, Faculty of Graduate Studies and Research, Somali National University, Mogadishu, Somalia; 3 Center for Epidemic Intelligence, Preparedness and Response, Mogadishu, Somalia; 4 Royal Veterinary College, University of London, London, United Kingdom

**Keywords:** fragile settings, health security, One Health, pandemic fund, preparedness financing, Somalia

## Abstract

The approval of Somalia’s proposal under the third call for proposals from the Pandemic Fund represents a major achievement in strengthening the country’s pandemic prevention, preparedness, and response (PPR) efforts. The award, totaling approximately $25 million from the Pandemic Fund and nearly US$120 million in co-financing and in-kind contributions from international partners and government sources, demonstrates growing international confidence in Somalia’s reform efforts and in its ability to implement multi-sectoral preparedness initiatives. Somalia is part of a priority group for the Pandemic Fund due to its fragile, conflict-affected situation, in which the risk of epidemics is amplified by institutional vulnerabilities. This paper discusses the strategic implications of Somalia’s Pandemic Fund award, outlines priority areas for investment, and explores how catalytic financing, if properly governed, could lead to systemic transformation.

## 1 Introduction

Pandemic preparedness has become a central pillar of global health security in the post-COVID-19 era, yet financing and implementation gaps continue to undermine equitable readiness in fragile and conflict-affected states (1). In response, the Pandemic Fund was created as a dedicated multilateral financing instrument to provide sustained, country-driven investments in surveillance, laboratory systems, workforce development, and emergency coordination, while incentivizing domestic ownership and partner co-investment (2). To date, the Fund has allocated nearly US $885 million across two funding rounds, leveraging over US $6 billion in co-financing from national and partner sources and supporting 75 countries globally (3).

Somalia’s health sector has evolved amid decades of insecurity, climate-related shocks, large-scale displacement, chronic underinvestment, and recurrent infectious disease outbreaks, including cholera, measles, and other epidemic-prone diseases (4, 5). These pressures have historically encouraged short-term, crisis-driven interventions rather than the sustained development of core preparedness functions, leaving surveillance coverage, diagnostic capacity, and emergency coordination mechanisms uneven nationwide. The health system remains severely under-resourced, with the Universal Health Coverage Service Coverage Index standing at 33.5 out of 100, compared to the Sub-Saharan African average of 42.5 (6). Similarly, the overall International Health Regulations (IHR) compliance score is 31 out of 100, reflecting weak surveillance systems, workforce shortages, and fragmented governance structures (7). The health workforce density is critically low, with only 0.11 clinicians per 1,000 population and 4.45 health workers per 1,000 population overall, both below the WHO-recommended threshold, further constraining service delivery and outbreak response capacity (8). Against this backdrop, securing support from the Pandemic Fund signals international confidence in Somalia’s reform trajectory and its ability to translate external financing into functional preparedness systems. National strategic frameworks increasingly emphasize health security reform and alignment with international preparedness standards; however, reliance on episodic emergency financing has limited institutional consolidation (9). Within this context, the Pandemic Fund award represents both recognition of ongoing reform efforts and an opportunity to institutionalize long-term preparedness capacities.

Somalia was awarded approximately US$25 million from the Pandemic Fund, which leveraged nearly US$100 million in co-financing from international partners and US$20 million in co-investment from the government’s own budget, bringing the total envelope to approximately US$145 million (10, 11). This scale distinguishes the initiative from prior preparedness projects that were typically limited in geographic scope or duration and positions the country to pursue nationally integrated reforms rather than isolated pilots. Somalia’s selection under a competitive call underscores international confidence in the technical credibility of its proposal, the feasibility of implementation arrangements, and the political commitment to multisectoral coordination. The proposal’s emphasis on integrated One Health systems, multisectoral coordination, and fragile-context preparedness likely contributed to its competitiveness under the Pandemic Fund’s strategic priorities.

## 2 Approach

This perspective draws on publicly available Pandemic Fund documents, Somalia’s national health-security and preparedness frameworks, and relevant global health-security literature to examine the strategic implications of Somalia’s Pandemic Fund award for strengthening pandemic prevention, preparedness, and response (PPR) systems. The manuscript synthesizes policy documents, peer-reviewed literature, and institutional perspectives related to fragile-state preparedness financing, One Health implementation, health-system resilience, and emergency preparedness governance. The analysis focuses on identifying priority investment domains, institutional opportunities, and implementation challenges associated with Pandemic Fund–supported interventions in Somalia. No primary data collection or human participant involvement was undertaken.

## 3 Strategic significance of the pandemic fund award

### 3.1 Endorsement of integrated one health approaches

Somalia’s proposal foregrounds a One Health orientation, integrating surveillance and response across human, animal, and environmental health interfaces. Such integration is essential in settings where zoonotic spillover risks, climate variability, and livelihood dependencies on livestock converge to shape epidemic emergence and propagation (12). Adopting One Health frameworks aligns with global best practices for PPR and reinforces multisectoral coordination that spans traditional institutional boundaries. Institutionalizing collaboration across ministries responsible for health, livestock, environment, and agriculture offers an opportunity to reduce long-standing silos and strengthen early-warning capacity.

### 3.2 Strengthening institutional capacity

Somalia has historically faced limitations in several core capacities under the International Health Regulations, including laboratory reach, workforce depth, and emergency-management systems. Somalia has articulated preparedness priorities through national action plans, follow-up to Joint External Evaluations, and the gradual establishment of Emergency Operations Centers (7). Pandemic Fund resources provide an opportunity to consolidate technical platforms, embed trained One Health officers and community teams in a gender-responsive manner, and standardize referral networks and quality-assurance mechanisms for priority pathogens. Success in a competitive Pandemic Fund call suggests that the proposed interventions were technically credible, aligned with international standards, and considered implementable despite contextual constraints.

### 3.3 Transition from reactive response to institutionalized preparedness

Historically, epidemic control in Somalia has relied heavily on humanitarian surge mechanisms mobilized during crises (13). The magnitude and multi-year horizon of the Pandemic Fund investment create conditions for a strategic rebalancing toward standing preparedness capacities, routine surveillance systems, decentralized laboratories, permanent emergency-operations platforms, and professionalized cadres of public-health responders.

### 3.4 Catalyzing multi-partner coordination

The Pandemic Fund’s model encourages alignment among national governments, development partners, and accredited implementing entities to reduce fragmentation and enhance coherence in preparedness investments (14). By situating financing within broader health systems agendas, including national health strategies and IHR core capacity improvements, Somalia’s award underscores improved strategic planning and accountability at institutional and governance levels.

**Figure 1:**
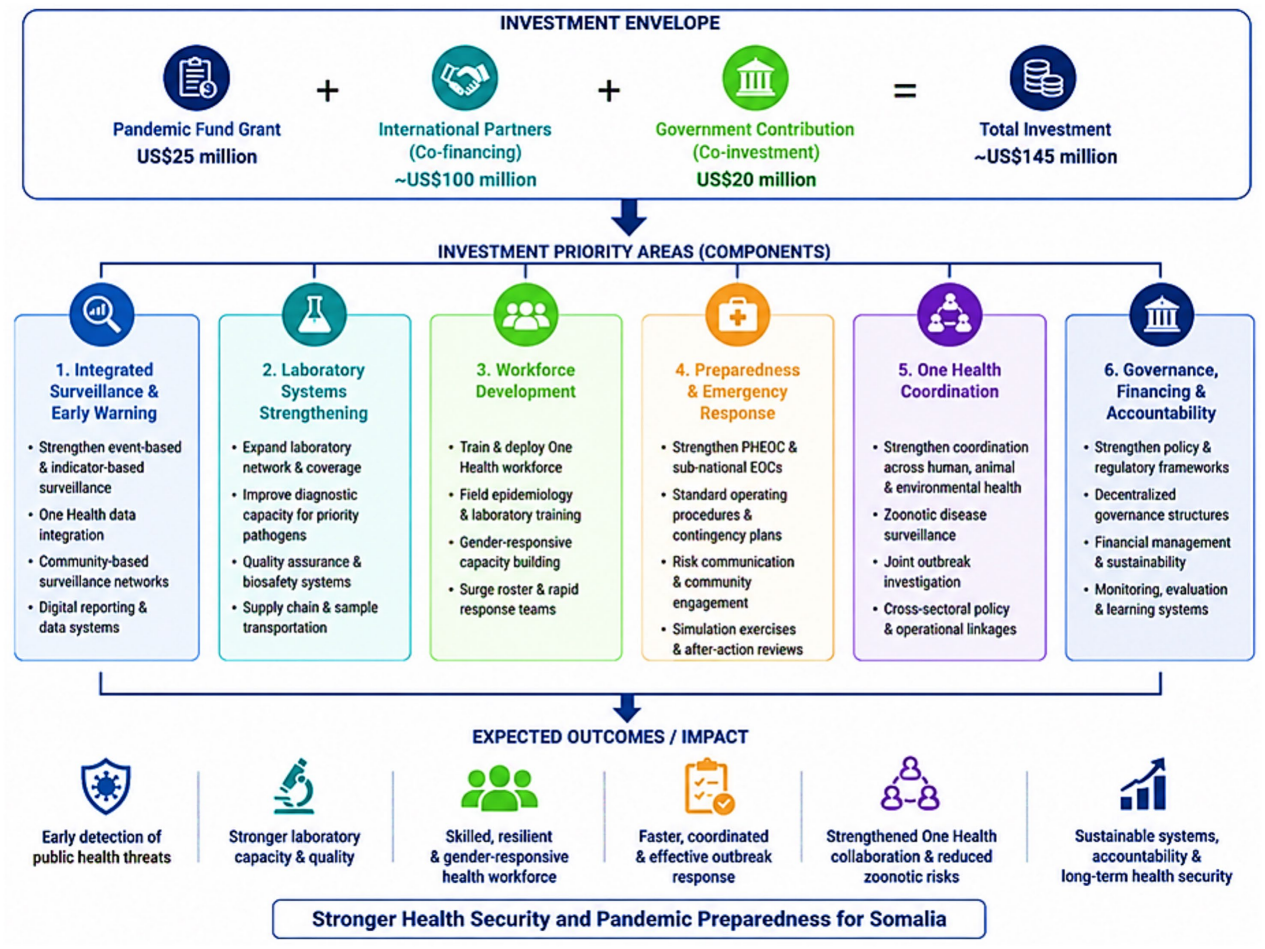
Pandemic fund investment pathway for strengthening Somalia’s PPR system. Conceptual framework illustrating financing flows, priority preparedness investment domains, multisectoral implementation pathways, and expected health-security outcomes in Somalia.

Figure 1 presents the conceptual framework illustrating financing flows, priority preparedness domains and investment pathways, multisectoral implementation pathways, and expected health-security outcomes in Somalia.

## 4 Priority investment domains

### 4.1 Integrated surveillance and early-warning systems

Strengthening indicator-based, event-based, and community-level surveillance systems constitutes a central pillar of the programme. Planned investments include enhancing digital reporting platforms, improving interoperability between human and animal health information systems, and expanding surveillance coverage in hard-to-reach and displacement-affected areas. Additional efforts focus on strengthening surveillance at points of entry, including monitoring of priority zoonotic diseases and antimicrobial resistance (AMR), as well as implementing a One Health Risk Communication and Community Engagement (RCCE) strategy. Activities also include strengthening multisectoral event verification and investigation, alongside improving data analysis and information-sharing systems. These measures are expected to reduce notification delays, improve situational awareness, and support evidence-informed decision-making at both national and sub-national levels.

### 4.2 Laboratory networks and diagnostic capacity

Diagnostic services in Somalia remain concentrated in a limited number of central facilities, constraining timely outbreak confirmation in peripheral regions. Pandemic Fund–supported investments aim to expand regional laboratory capacity, strengthen specimen referral and transport systems, institutionalize quality assurance mechanisms, and enhance biosafety and biosecurity standards. The programme also prioritizes establishing cross-sectoral laboratory networks linking human and animal health sectors. These reforms are critical for decentralized detection, improved antimicrobial resistance monitoring, and more effective outbreak response.

### 4.3 Workforce development and retention

Human resources remain central to preparedness and response. The programme prioritizes training in field epidemiology, laboratory sciences, emergency operations management, and community-based surveillance, alongside the development of One Health coordination cadres. It also emphasizes building and maintaining community-based health worker networks and developing surge response capacity to support rapid outbreak detection and response. Embedding trained personnel within public-sector systems and introducing retention strategies will be essential to sustaining competencies and reducing reliance on external surge mechanisms.

## 5 Opportunities for system-level transformation

### 5.1 Federal and sub-national alignment

Somalia’s federal governance system presents coordination challenges but also opportunities for decentralized preparedness implementation. Strategic investment in sub-national institutions and community-engagement platforms can strengthen Centre–periphery linkages, provided institutional roles and mandates are clearly articulated and supported by transparent governance arrangements.

### 5.2 Integrating climate risk and health security

Climate-related shocks, particularly droughts and floods, are major drivers of displacement and epidemic vulnerability. Incorporating preparedness investments with disaster-risk-reduction and anticipatory-action triggers into health-security planning could improve preparedness for climate-sensitive outbreaks and position Somalia as a regional example of integrated resilience planning.

### 5.3 Strengthening accountability and stewardship

Effective stewardship mechanisms, including transparent performance monitoring, fiduciary oversight, and parliamentary engagement, are critical to ensuring that Pandemic Fund resources translate into durable system functions. Strengthening governance alongside technical capacity will be central to maintaining public trust and donor confidence.

## 6 Risk considerations and implementation challenges

Despite its promise, the initiative entails substantial risks characteristic of fragile settings, including insecurity in certain regions, workforce attrition, procurement bottlenecks, limited absorptive capacity, and the potential proliferation of parallel partner systems if coordination falters (14). Rapid scale-up could exacerbate centre–periphery tensions or strain nascent administrative structures unless reforms are carefully sequenced.

Mitigation strategies should therefore prioritize phased implementation, harmonized partner coordination mechanisms, incentives for staff retention, strengthened fiduciary oversight, and sustained investment in local governance and community-engagement structures. Integrating preparedness activities into routine service delivery and aligning external financing with national strategies will be essential to sustaining gains beyond the project period.

## 7 Implications for global health-security financing

Somalia’s success offers an instructive case for pandemic-preparedness financing in fragile and conflict-affected settings. Demonstrable improvements, such as reduced outbreak-detection times, sustained laboratory performance, and faster emergency activation, would strengthen the argument for directing a larger share of global PPR resources toward high-risk environments rather than concentrating investments in already resilient systems (15, 16). Systematic documentation of Somalia’s implementation experience through operational research could further inform future Pandemic Fund rounds, shaping proposal design, fiduciary-risk mitigation strategies, and incentives for cross-sectoral collaboration.

## 8 Conclusion

Somalia’s Pandemic Fund award, supported by approximately US $145 million in combined financing, marks a pivotal juncture in the country’s health-security trajectory. Its ultimate significance will depend on whether these resources yield enduring institutional capacity: surveillance systems that function between crises, laboratories that consistently meet quality standards, a skilled workforce embedded within government, and emergency-management platforms capable of rapid activation. If effectively implemented, Somalia’s programme could serve as a model for how fragile states translate large-scale financing into integrated, multisectoral, and institutionalized preparedness systems. Sustained political commitment, domestic ownership, and coordinated partner engagement will be essential to ensuring long-term sustainability beyond the Pandemic Fund implementation period.

## Data Availability

The original contributions presented in the study are included in the article/supplementary material, further inquiries can be directed to the corresponding author.
