# Correction: Strengthening pandemic prevention, preparedness, and response in Somalia: strategic implications of health security financing

**DOI:** 10.3389/fpubh.2026.1930879

**Published:** 2026-07-23

**Authors:** Abdullahi Ahmed Tahlil, Nor Ali Mohamoud, Ali Haji Adam, Abdinasir Yusuf Osman

**Affiliations:** 1 Ministry of Health and Human Services, Federal Government of Somalia, Mogadishu, Somalia; 2 Department of Public Health, Faculty of Graduate Studies and Research, Somali National University, Mogadishu, Somalia; 3 Center for Epidemic Intelligence, Preparedness and Response, Mogadishu, Somalia; 4 Royal Veterinary College, University of London, London, United Kingdom

**Keywords:** fragile settings, health security, One Health, pandemic fund, preparedness financing, Somalia

Affiliations ^2^Department of Public Health, Faculty of Graduate Studies and Research, Somali National University, Mogadishu, Somalia and ^3^Center for Epidemic Intelligence, Preparedness and Response, Mogadishu, Somalia were omitted from the paper. These affiliations have now been added for author Abdullahi Ahmed Tahlil.

The original version of this article has been updated.

